# Insights from genomes and genetic epidemiology of SARS-CoV-2 isolates from the state of Andhra Pradesh

**DOI:** 10.1017/S0950268821001424

**Published:** 2021-08-03

**Authors:** Pallavali Roja Rani, Mohamed Imran, J. Vijaya Lakshmi, Bani Jolly, S. Afsar, Abhinav Jain, Mohit Kumar Divakar, Panyam Suresh, Disha Sharma, Nambi Rajesh, Rahul C. Bhoyar, Dasari Ankaiah, Sanaga Shanthi Kumari, Gyan Ranjan, Valluri Anitha Lavanya, Mercy Rophina, S. Umadevi, Paras Sehgal, Avula Renuka Devi, A. Surekha, Pulala Chandra Sekhar, Rajamadugu Hymavathy, P.R. Vanaja, Vinod Scaria, Sridhar Sivasubbu

**Affiliations:** 1 Kurnool Medical College, Kurnool, Andhra Pradesh518002, India; 2 CSIR Institute of Genomics and Integrative Biology (CSIR-IGIB), Delhi110025, India; 3 Academy of Scientific and Innovative Research (AcSIR), Ghaziabad201002, India

**Keywords:** COVID-19, SARS-CoV-2, genetic epidemiology, Andhra Pradesh

## Abstract

Coronavirus disease 2019 (COVID-19) emerged from a city in China and has now spread as a global pandemic affecting millions of individuals. The causative agent, severe acute respiratory syndrome coronavirus 2 (SARS-CoV-2), is being extensively studied in terms of its genetic epidemiology using genomic approaches. Andhra Pradesh is one of the major states of India with the third-largest number of COVID-19 cases with a limited understanding of its genetic epidemiology. In this study, we have sequenced 293 SARS-CoV-2 genome isolates from Andhra Pradesh with a mean coverage of 13324X. We identified 564 high-quality SARS-CoV-2 variants. A total of 18 variants mapped to reverse transcription polymerase chain reaction primer/probe sites, and four variants are known to be associated with an increase in infectivity. Phylogenetic analysis of the genomes revealed the circulating SARS-CoV-2 in Andhra Pradesh majorly clustered under the clade A2a (20A, 20B and 20C) (94%), whereas 6% fall under the I/A3i clade, a clade previously defined to be present in large numbers in India. To the best of our knowledge, this is the most comprehensive genetic epidemiological analysis performed for the state of Andhra Pradesh.

The emergence of coronavirus disease 2019 (COVID-19) as a global pandemic has necessitated approaches to understand the evolution and transmission of severe acute respiratory syndrome coronavirus 2 (SARS-CoV-2). Genome sequencing has emerged as one of the widely used approaches to understand the genetic epidemiology of SARS-CoV-2 [1]. The availability of the complete genome of the pathogen early in the epidemic and subsequent application of genomics on a global and unprecedented scale has provided an immense opportunity to trace the introduction, spread and genetic evolution of the SARS-CoV-2 across the globe [2].

India is now a major country affected by COVID-19 with over 10 million people affected since the initial introduction of SARS-CoV-2 into the country in 2020 and subsequent introductions through travellers across major cities. These include states with significantly large populations and air travellers such as Andhra Pradesh which has a population of 49 million, with an estimated 1.5–2 million people who are part of the diaspora spread across the world. Although a number of genomes have been sequenced from different states in India [3], there is a paucity of genomic data and genetic epidemiology of SARS-CoV-2 isolates from the state of Andhra Pradesh which motivated us to study the genomes from this state in detail. In the current study, we report a total of 293 SARS-CoV-2 genomes from the state of Andhra Pradesh. To the best of our knowledge, this is the first comprehensive report of the genetic epidemiology and evolution of SARS-CoV-2 from the state of Andhra Pradesh.

The study is in compliance with relevant laws and institutional guidelines and in accordance with the ethical standards of the Declaration of Helsinki and approved by Institutional Human Ethics Committee (RC. No. 03/IHC/kmcknl/2020, dated 03/08/2020). The patient consent has been waived by the ethics committee. RNA samples isolated from nasopharyngeal/oropharyngeal swabs of patients from a tertiary care teaching hospital (Kurnool Medical College) were used in the study. RNA isolation was performed using GenoSens SARS-CoV-2 PCR Viral RNA extraction reagents and the Truprep system (Molbio Diagnostics) for COVID-19. All samples were confirmed by multiplex reverse transcription polymerase chain reaction (RT-PCR).

In total, 143 726 samples were tested between 21 April to 5 August and 10 073 samples were identified as COVID-19 positive. In total, 293 samples collected between 27 June 2020 and 3 August 2020 were considered for viral genome sequencing. Library preparation and sequencing was performed as per the COVIDSeq protocol (Illumina, USA) as described in a previous study [4]. The samples were sequenced in technical replicates.

We followed a previously published protocol for data analysis [5]. Briefly, raw FASTQ files underwent quality control and adapter trimming using Trimmomatic (version 0.39) [6]. The Wuhan-Hu-1 (NC_045512.2) genome was used as the reference. Replicate files were independently aligned and merged. Genomes with ≥99% coverage and ≤5% unassigned nucleotides were processed for variant calls. Genetic variants were annotated by ANNOVAR [7] using custom database tables for annotating the SARS-CoV-2 genome. Filtered variants were systematically compared with other viral genomes deposited in the Global Initiative on Sharing All Influenza Data (GISAID). Genomes from the GISAID were aligned with the reference genome using EMBOSS [8] and variants were called using SNP-Sites [9]. Only genomes with an alignment percentage of ≥99% and degenerate bases ≤5% were used for comparative analysis. This accounts for a total of 45 830 high-quality genome sequences submitted until 26 September 2020 (Supplementary Table S1).

Phylogenetic analysis was performed following the Nextstrain protocol for analysis of SARS-CoV-2 genomes using the genomes sequenced in this study and the dataset of 3058 genomes from India deposited in the GISAID (Supplementary Table S2) [10–12]. Lineages were assigned to the genomes using the Phylogenetic Assignment of Named Global Outbreak LINeages (pangoLEARN version 2020-07-20) package [13]. Genetic variants in the genomes sequenced were mapped against RT-PCR primer/probe sites used in the molecular detection of SARS-CoV-2 [14] and with other variants associated with functional consequences in the viral genome which were compiled from the published studies and article pre-prints.

A total of 200 μl of VTM (Himedia, Mumbai) with throat swab samples were used to extract the viral RNA from subjects with symptoms of COVID-19 infection. A total of 10 μl of viral RNA was used for RT-PCR detection. Target genes, including ORF1ab gene, RNA-dependent RNA polymerase (RdRP gene), nucleocapsid protein (N gene) and envelope protein (E gene) were simultaneously amplified and tested during the real-time PCR assay. Confirmed SARS-CoV-2 RNA extracts with Ct values 22–28 were further processed for whole-genome sequencing.

In total, 293 SARS-CoV-2 genomes were sequenced with a mapping percentage of 97.27% and 13324X coverage (Supplementary Table S3). In total, 276 samples having genome coverage ≥99% and ≤5% unassigned nucleotides were further processed for variant calling and consensus sequence generation.

The reference-based assembly generated a total of 615 unique genetic variants. Out of these, 564 variants having read frequencies ≥50% were considered for the comparative analysis. The distribution of the variants in the genomes and their annotations are summarised in Supplementary Figure S1. Of the total variants considered for comparative analysis, 72 were found to span the spike protein region. We identified four genetic variants in the S gene which have been previously reported to be involved in increased infectivity through experimental validation [15–22]. These mutations include 23403:A>G (D614G) and three co-occurring mutations 23403A>G+ 21575C>T (D614G + L5F), 23403A>G+ 24368G>T (D614G + D936Y), 23403A>G+ 24378C>T (D614G + S939F), having a frequency of 94.20%, 0.725%, 5.435% and 0.362%, respectively, in the 276 genomes analysed in this study. The sequence variant N440K spanning the receptor-binding domain of SARS-CoV-2 spike protein was found in 92 samples out of the 276 genomes analysed in this study. N440K is one of the hotspot residues involved in viral immune escape mechanisms and has been found to be resistant to a range of monoclonal antibodies including C135 and REGN10987 [23–25]. This variant has also been reported in a case of SARS-CoV-2 reinfection, including one report from within the state of Andhra Pradesh, and has been recently reported to have higher infective fitness compared to the prevalent A2a clade [26–28]. A total of 145 variants were annotated as deleterious by SIFT [29] whereas 18 genetic variants mapped to diagnostic RT-PCR primer/probe sites (Supplementary Table S4). A total of 42 and 421 variants were predicted to map to potential B and T cell epitopes, respectively (Supplementary Table S5).

Phylogenetic analysis was conducted for the dataset of 3033 SARS-CoV-2 genomes from India including 276 genomes from this study and the genome Wuhan/WH01 (EPI_ISL_406798) as the root. Out of 276 genomes, 260 genomes (94%) clustered under the clade A2a (20A, 20B and 20C) whereas 16 were under clade I/A3i (6%), a distinct cluster of genomes previously reported from India [30] (Fig. 1a and 1b). The dominant lineages for the 276 genomes, as assigned by PANGOLIN, were B.1.113 (*n* = 129) and B.1 (*n* = 95) as compared to other Indian genomes where B.1.1.32 and B.6 were dominant whereas B.1 and B.1.1 lineages were dominant for genomes in the global dataset. Five and one genomes were assigned lineages B.1.112 and B.1.104, respectively, which have not been previously reported for the genomes from India (Fig. 1c).

**Fig. 1.**
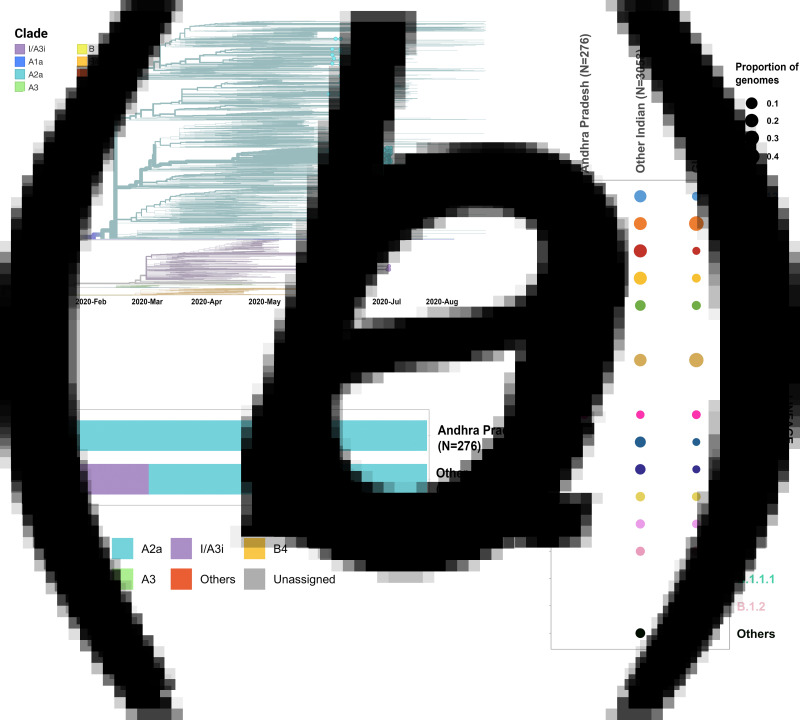
(a) Time-resolved phylogenetic reconstruction of 3033 SARS-CoV-2 genomes from India. In total, 276 genomes from this study are highlighted. (b) Proportion of clades in the 276 genomes from Andhra Pradesh and other genomes from India. (c) Distribution of PANGOLIN lineages in the genomes in this study in comparison with other genomes from India and across the world.

The two earlier genomes from Andhra Pradesh (EPI_ISL_436440 and EPI_ISL_435089) [31] sampled in the months of March and April, respectively, cluster under clade I/A3i. Genomes from the neighbouring state of Telangana also show a dominant prevalence of clade I/A3i (86% of all sequenced genomes) in the initial days of the pandemic (March–April 2020) and an increased representation of clade A2a in later months with 26.6% of all sequenced genomes belonging to clade I/A3i [30]. The genomes sequenced in this study show a prevalence of clade A2a in Andhra Pradesh which suggests a shift in clade dominance for this region. From the 263 genomes that cluster under clade A2a, a majority of the genomes (*n* = 171) were observed to fall under a distinct sub-cluster that has been previously reported for genomes from Gujarat [32]. The cluster is characterised by an S194L (C28854T) mutation in the nucleocapsid protein of the virus, a mutation that was found to be significantly associated with disease mortality in Gujarat [32]. One genome from this study (CS0804) also forms a polytomy with other samples from the neighbouring state of Telangana in the phylogenetic tree of Indian genomes, which could be suggestive of multiple, simultaneous divergence events although further data and analysis would be needed to confirm this hypothesis reliably.

Put together, our analysis using the COVIDSeq approach and downstream data analysis has provided detailed insights into the genetic epidemiology and evolution of SARS-CoV-2 isolates in the state of Andhra Pradesh. A total of 564 high-quality unique genetic variants were identified, out of which 15 variants are novel. Extensive analysis of the functional consequences of the filtered variants has provided insights into the impact of these genetic variants in current diagnostic practices.

Phylogenetic analysis of the genomes highlights the potential shift in clade dominance from clade I/A3i to A2a in Andhra Pradesh, a trend also observed in the neighbouring state of Telangana. Lineages B.1.112 and B.1.104 were also reported for the first time from Indian genomes.

In conclusion, our study highlights the utility of whole-genome sequencing to study the genetic landscape and evolution of SARS-CoV-2 isolates in major states such as Andhra Pradesh and emphasises the use of such scalable technologies to gain better and timely insights into epidemics.

## Data Availability

The data that support the findings of this study are available at NCBI Short Read Archive with Project ID PRJNA662193 with accession IDs from SAMN16707355 to SAMN16707555. Dataset of the remaining samples is available at NCBI Short Read Archive with Project ID PRJNA655577.

## Supplementary material

For supplementary material accompanying this paper visit http://dx.doi.org/10.1017/S0950268821001424. click here to view supplementary material
