# Supplementary figures and images for: Insights from genomes and genetic epidemiology of SARS-CoV-2 isolates from the state of Andhra Pradesh
Source: Epidemiol Infect. 2021 Aug 3;149:e181. doi: 10.1017/S0950268821001424 (PMC8367868; doi:10.1017/S0950268821001424)

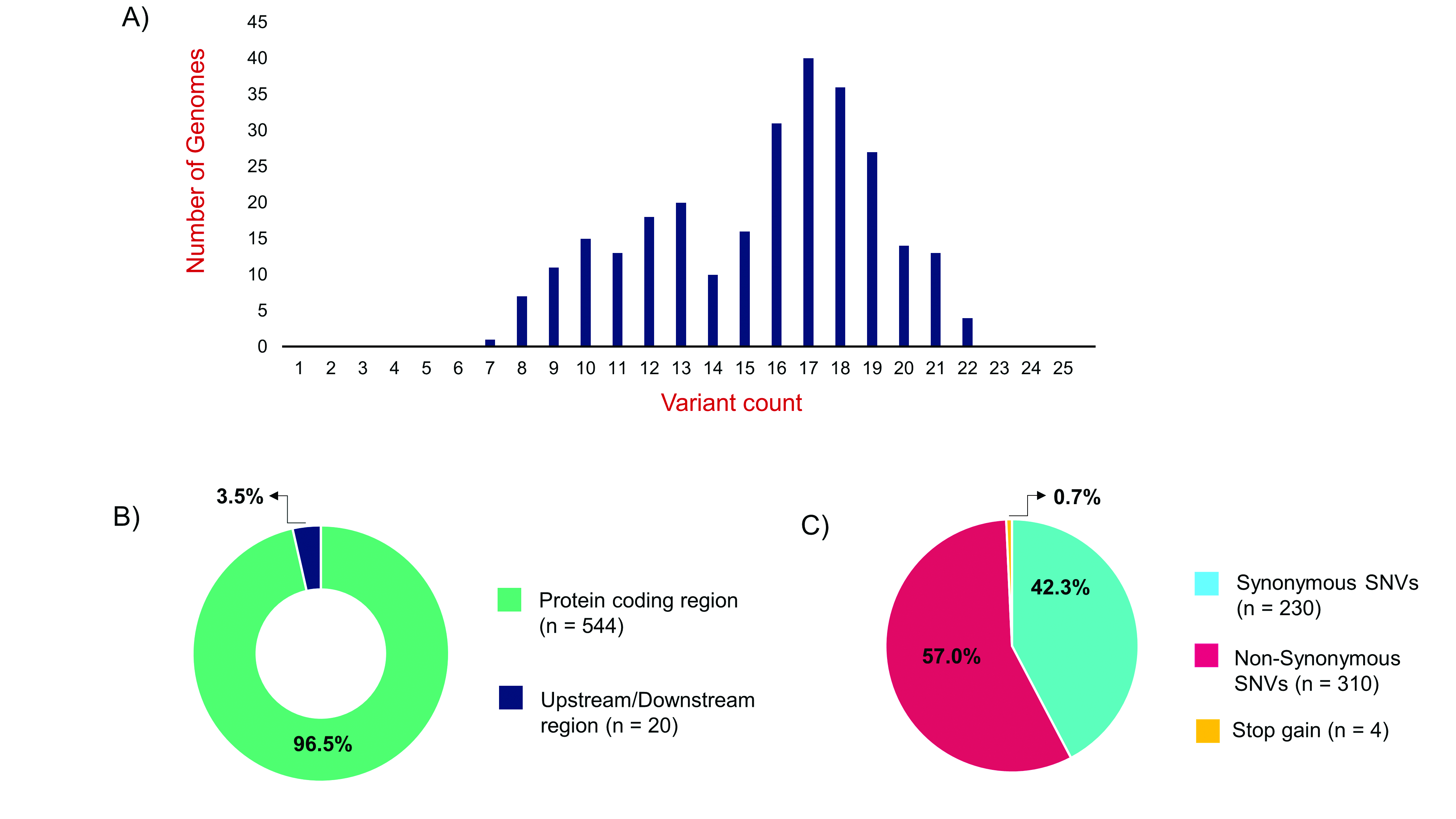

Supplement: Supplementary file 1 [file hygsup.zip › S0950268821001424sup001.tif]
